# Complete Genome Sequence of *Escherichia coli* O145:NM Bacteriophage vB_EcoM_AYO145A, a New Member of O1-Like Phages

**DOI:** 10.1128/genomeA.00539-15

**Published:** 2015-06-18

**Authors:** Jiaying Wang, Yan D. Niu, Jinding Chen, Tim A. McAllister, Kim Stanford

**Affiliations:** a College of Veterinary Medicine, South China Agricultural University, Guangzhou, Guangdong, China; b Agriculture and Agri-Food Canada, Lethbridge, Alberta, Canada; c Alberta Agriculture and Rural Development, Lethbridge, Alberta, Canada

## Abstract

Previously, bacteriophage vB_EcoM_AYO145A, which lyses Shiga toxin-producing *Escherichia coli* O145:NM, was classified as an O1-like virus of the *Myoviridae* family. Here, we report the complete genome sequence of this phage and a comparative genomic analysis with other known O1-like phages.

## GENOME ANNOUNCEMENT

*Escherichia coli* O145 is one of the top six non-O157 Shiga toxin-producing *E. coli* (STEC) strains and has been associated with multiple foodborne outbreaks worldwide (1). Control measures for STEC O145 have been limited, and bacteriophages offer considerable potential for the biocontrol of STEC in the environment (2). Previously, phage AYO145A, a member of the O1-like phages, lysed all STEC O145:NM strains (3). The objective of this study was to describe the genome sequence of this phage, the first complete genome of a STEC O145 phage.

Phage AYO145A was isolated from a cattle transport trailer in Alberta, Canada (3). Phage DNA was extracted from the CsCl-purified phage lysates using the SDS-proteinase K protocol (4). The CsCl-purified phage DNA was sequenced using GS-FLX+ (Laval University, Québec, Canada). Sequencing reads (24,907) with ~85-fold coverage were assembled with the gsAssembler module of Newbler version 2.5.3. The genome was annotated, as described previously (5). Pairwise nucleotide and amino acid sequence identities were calculated by EMBOSS Stretcher (6) and Align (7), respectively. Clustal Omega (8) was used to align the amino acid sequences of tail fiber proteins.

The AYO145A genome consists of 87,372 bp of double-stranded DNA (dsDNA), with a G+C content of 39%. A total of 131 coding sequences (CDSs) and 20 tRNAs, which encode 16 amino acids, were identified. Furthermore, 16 rho-dependent terminators and 16 promoters recognized by host RNA polymerase were identified. Position-specific iterated (PSI)-BLAST searches indicated that the protein products of 128 CDSs were similar to those of O1-like phages. None of the predicted proteins exhibited homology toward virulence factors, integration-related proteins, or antibiotic resistance determinants. Comparative genomic analysis revealed that AYO145A was collinear with Felix O1 (83.8% identity) (9) and wV8 (87.2%) (10) but distantly related to phiEa21-4 (51.8%) (11). Computational analysis of CoreGenes (12) showed that AYO145A shares 119 (90.8%), 124 (88.6%), 118 (82.5%), and 66 (55.9%) homologues with phages Felix O1, wV8, and phiEa21-4, respectively. Compared to phages Felix O1 and wV8, the genome of AYO145A mainly differs in regions of 6.4 to 6.8, 51 to 54.7, and 64.2 to 64.6 kbp, which include CDSs for two adjacent tail fiber proteins and a DNA polymerase. However, further analysis of the amino acid sequences of these CDSs showed that this DNA polymerase is well conserved among phages AYO145A, Felix O1, and wV8 (>91.9% amino acid identity [ID]). Not surprisingly, the amino acid sequence of each tail fiber protein from AYO145A differs from its counterparts in Felix O1 (68.8 to 76.7% ID) and wV8 (60.3 to 78% ID). Moreover, the tail fiber protein (788 amino acids [aa]) alignment of these three phages displayed four regions of similarity separated by regions of dissimilarity, with both the C and N termini conserved.

### Nucleotide sequence accession number.

The complete genome sequence of *E. coli* phage vB_EcoM_AYO145A has been deposited in GenBank under the accession no. KR014248.
